# Passivation and Interlayer Effect of Zr(i-PrO)_4_ on Green CuGaS_2_/ZnS/Zr(i-PrO)_4_@Al_2_O_3_ and Red CuInS_2_/ZnS/Zr(i-PrO)_4_@Al_2_O_3_ QD Hybrid Powders

**DOI:** 10.1186/s11671-022-03741-0

**Published:** 2022-11-07

**Authors:** Minji Ko, Soyeon Yoon, Yun Jae Eo, Keyong Nam Lee, Young Rag Do

**Affiliations:** Department of Chemistry, Kookmin University, Seoul, 02707 Republic of Korea

**Keywords:** I–III–VI quantum dots, Hydrolysis, Surface passivation, Stability, QD doping

## Abstract

Broadband emissive I–III–VI quantum dots (QDs) are synthesized as efficient and stable I–III–VI QDs to be used as eco-friendly luminescent materials in various applications. Here, we introduce the additional passivation of zirconium isopropoxide (Zr(i-PrO)_4_) to improve the optical properties and environmental stability of green-emitting CuGaS_2_/ZnS (G-CGS/ZnS) and red-emitting CuInS_2_/ZnS (R-CIS/ZnS) QDs. The photoluminescence quantum yield (PLQY) of both resultant Zr(i-PrO)_4_-coated G-CGS/ZnS and R-CIS/ZnS QDs reaches similar values of ~ 95%. In addition, the photostability and thermal-stability of G-CGS/ZnS/Zr(i-PrO)_4_ and R-CIS/ZnS/Zr(i-PrO)_4_ QDs are improved by reducing the ligand loss via encapsulation of the ligand-coated QD surface with Zr(i-PrO)_4_. It is also proved that the Zr(i-PrO)_4_-passivated interlayer mitigates the further degradation of I-III-V QDs from ligand loss even under harsh conditions during additional hydrolysis reaction of aluminum tri-sec-butoxide (Al(sec-BuO)_3_), forming easy-to-handle G-CGS/ZnS and R-CIS/ZnS QD-embedded Al_2_O_3_ powders. Therefore, the introduction of a Zr(i-PrO)_4_ complex layer potentially provides a strong interlayer to mitigate degradation of I–III–VI QD-embedded Al_2_O_3_ hybrid powders as well as passivation layer for protecting I–III–VI QD.

**Supplementary Information:**

The online version contains supplementary material available at 10.1186/s11671-022-03741-0.

## Introduction

Recently, eco-friendly I–III–VI quantum dots (QDs) have been studied intensively in an effort to prepare broadband emissive QD materials for bio-applications and for lighting with high color rendering index (CRI) (> 90) [1–5]. However, QD emission characteristics and material stability levels remain inferior to those of environmentally toxic Cd-based II-VI chalcogenide QDs. Hence, improved photoluminescence quantum yields (PLQYs) and enhanced material stability of I–III–VI QDs are required for their application to various lighting and bio-applications. To date, the record high PLQYs of I–III–VI QDs have already exceeded 90% in the solution form of QD colloids dispersed in an organic solvent [6, 7]. Occasionally, as-prepared I–III–VI QD solutions are not sufficiently stable to be applied to lighting devices or bio-applications due to the limited UV and thermal stability of I–III–VI QDs during the transforming process of QD powders and post-coating process of oxide encapsulants [8, 9].

Surface passivation of QDs is a facile process that can be utilized during the synthesis of I–III–VI QDs for green and red (GR) color-converting materials in white LEDs [10–16]. It is well known that the ligand passivation of QDs can form a protective layer to improve the material stability of QDs against environmental attacks, such as those by light, humidity, and operation heat [17–20]. However, it is a critical issue that organic ligands quickly degrade under various environmental conditions, such as high temperature, intensive UV irradiation, and high humidity. To solve this issue, various additional surface passivation or encapsulation processes were introduced at defects sites on the QD surface to reduce ligand loss. Among them, the mitigation band gap between the core and shell structure is the one of the ways to enhance surface passivation [6, 12]. Introduction of a polymer matrix such as polyvinylpyrrolidone (PVP), polymethylmethacrylate (PMMA), and polyvinyl alcohol (PVA) is an additional way to improve surface passivation [13, 21]. Additional passivation was performed to maintain the high PLQY in the QD solution form under various environmental conditions [21–23]. Furthermore, metal–organic frameworks (MOFs) were developed as a method to enhance the stability of QDs [24]. As previously reported, the hydrolysis reaction of metal alkoxide precursors is a well-known and facile encapsulation process to embed QDs into an inorganic–organic matrix powder [22, 23, 25]. In most hydrolysis reactions of metal alkoxides, acid and base catalysts are used to accelerate hydrolysis reactions, and a small amount of water is necessary to complete the hydrolysis reactions. Unfortunately, both acid/based catalysts and water can cause photoluminescent quantum yield (PLQY) degradation of nearly all types of QDs during hydrolysis [26, 27]. Nonetheless, certain easy synthetic processes, including the Stöbber reaction [28–31] can enable most hydrolysis reactions to produce oxide encapsulated QD powders via inorganic polymerization and gelation and hydrolysis processes [11, 32–36]. Meanwhile, Prof. Yang’s group introduced a Zr-alkoxide that can be used as secondary passivation material for narrow-band InP/ZnSeS/ZnS-based QDs to improve environmental stability [37, 38]. However, there have been no detailed reports on secondary passivation effects of metal alkoxides to mitigate the degradation of eco-friendly I–III–VI QDs and I–III–VI QD-embedded oxide powders during simple hydrolysis reaction.

In this study, we enhanced the stability of both G-CGS/ZnS and R-CIS/ZnS QDs in two steps involving a secondary ligand and aluminum tri-sec-butoxide (Al(sec-BuO)_3_) [14, 22, 25]. Due to the introduction of zirconium isopropoxide (Zr(i-PrO)_4_) as a secondary ligand, the stability of both G-CGS/ZnS and R-CIS/ZnS QDs primarily increased. We further introduced Al(sec-BuO)_3_ as an effective precursor for use in fast hydrolysis reactions without requiring acid/base catalysts or water. So, G-CGS/ZnS and R-CIS/ZnS QD-embedded Al_2_O_3_ hybrid powders were synthesized by fast hydrolysis reaction with a mixture solution of Al(sec-BuO)_3_ and Zr(i-PrO)_4_-decorated I–III–VI QDs in toluene. We also analyzed the degree to which Zr(i-PrO)_4_ secondary ligands protect degradation of I–III–VI QDs during hydrolysis reaction. Finally, the passivation effect of Zr(i-PrO)_4_ secondary ligand was investigated by comparing the optical properties of two down-converted white LEDs (DC-WLEDs), which include G-CGS/ZnS and R-CIS/ZnS QDs@Al_2_O_3_ QD hybrid powders and G-CGS/ZnS and R-CIS/ZnS/Zr(i-PrO)_4_@Al_2_O_3_ QD hybrid powders. We confirmed that secondary ligand passivation such as a coating of Zr(i-PrO)_4_ not only improves the environmental stability of the eco-friendly I–III–VI QDs itself, but also mitigates further degradation of I–III–VI QDs during additional inorganic coating and device fabrication processes [25, 39].

## Experimental

### Materials

Copper(I) iodide (CuI, 99.999%, Aldrich), gallium(III) iodide (GaI_3_, 99.99%, Aldrich), indium(III) acetate (In(ac)_3_, 99.99%, Aldrich), sulfur (S, 99.98%, Aldrich), zinc acetate dihydrate (Zn(ac_2_, reagent grade, Aldrich), zinc stearate (10 − 12% Zn basis, Aldrich), zirconium(IV) propoxide solution (Zr(i-PrO)_4_, 70 wt% in 1-propanol, Aldrich), (oleylamine (OLA, 70%, Aldrich), 1-dodecanethiol (DDT, 98%, Aldrich), oleic acid (OA, 90%, Aldrich), 1-octadecene (ODE, 90%, Aldrich), aluminum-tri-sec-butoxide (Al(O-sec-Bu)_3_, 97%, Aldrich), UV-curable polymer (NOA 61, Norland Products, Inc.) and cup-type of InGaN LED (λ_max_ = 450 nm, Dongbu LED Co. Ltd., Inc.) were utilized.

### Synthesis of Red CIS/ZnS and CIS/ZnS/Zr(i-PrO)_4_ QDs

To synthesize the CIS/ZnS QDs, 0.125 mmol of CuI, 0.5 mmol of In(ac)_3_, 0.5 mL of DDT and 5 mL of OLA were loaded into a three-necked flask. The loaded precursors were supplied with N_2_ gas for 15 min at room temperature. After N_2_ purging, 0.2 mmol of sulfur dissolved in 2 mL of ODE was rapidly injected into the three-necked flask at 140 °C for three minutes. For the first shelling process, we injected 8 mL of a prepared Zn stock solution consisting of 5.3 mmol of Zn(ac)_3_ dissolved in 2.7 mL of ODE and 5.3 mL of OA at 240 °C for 30 min. For the second shelling process, 8 mL of the prepared Zn stock solution (5.3 mmol of zinc stearate) dissolved in 2.7 mL of DDT and 5.3 mL of ODE was loaded into the reaction pot at 240 °C for two hours. For the in-situ-treatment of Zr(i-PrO)_4_ with the synthesized CIS/ZnS QDs, 1 mL of the Zr(i-PrO)_4_ was injected into the reaction solution at 240 °C for 30 min. The synthesized QD solution was centrifuged for purification and dispersed in hexane.

### Synthesis of Green CGS/ZnS and CGS/ZnS/Zr(i-PrO)_4_ QDs

To synthesize the CGS/ZnS QDs, 0.125 mmol of CuI, 0.75 mmol of GaI_3_, 1.0 mmol of S, 1.5 mL of DDT and 5 mL of OLA were loaded into a three-necked flask. The loaded precursors were purged with N_2_ gas for 15 min at room temperature. The reaction flask was quickly heated to 240 °C and reacted for 5 min to grow the core. For the shelling process, we utilized three sequential steps. For the first shelling process, we injected 12 mL of a prepared Zn stock solution consisting of 8 mmol of Zn(ac)_3_ dissolved in 4 mL of ODE and 8 mL of OA at 240 °C for an hour. Second, we injected 8 mL of a prepared Zn stock solution consisting of 4 mmol of Zn(ac)_3_ dissolved in 2 mL of ODE, 2 mL of DDT and 4 mL of OA at 240 °C for 30 min. Lastly, we injected a Zn stock solution consisting of 4 mmol of Zinc stearate dissolved in 4 mL of DDT and 4 mL of ODE at 250 °C for two hours. 1 mL of Zr(i-PrO)_4_ was injected into the growth solution at 250 °C for 30 min to initiate in-situ-treatment of Zr(i-PrO)_4_ on the surface of synthesized CGS/ZnS QDs. The synthesized QD solution was dispersed in hexane after the purification.

### Fabrication of Al_2_O_3_ Encapsulated Red and Green QD Hybrid Powders

The purified QDs were diluted to an optical density of 1.4 (at 450 nm). We prepared a 30 wt% Al(O-sec-Bu)_3_ solution that dissolved in toluene. We prepared Al-QD stock solution in which the diluted QDs were added to the prepared 30 wt% Al(O-sec-Bu)_3_ solution at a volume ratio of 2:1. Al_2_O_3_ encapsulated red and green QD hybrid powders were fabricated through ex-situ hydrolysis reaction of prepared Al-QD stock solution under ambient moisture at room temperature.

### Fabrication of Green–Red QD-Embedded Al_2_O_3_ Hybrid Powder-Based Single-Package WLED

The obtained green and red QD-embedded Al_2_O_3_ powders were mixed with the UV-curable binder NOA 61 to fabricate a single-package WLED. An appropriate amount of green and red QD powder/NOA 61 mixture was dropped onto a cup-type InGaN blue LED, which was used as an excitation source. The InGaN blue LED to which green and red QD powder/NOA 61 was then exposed to UV light (at 365 nm) for 30 min to harden the green and red QD/NOA 61 mixture. The obtained green–red QD-embedded Al_2_O_3_ hybrid powder-based single-package was realized as a 6,500 K white down-converted LED at an applied current of 60 mA.

### Characterization

The absorbance and PL emission spectra of the synthesized red CIS/ZnS, CIS/ZnS/Zr(i-PrO)_4_ and green CGS/ZnS, CGS/ZnS/Zr(i-PrO)_4_ QDs were measured with a UV–visible spectrometer (Lambda 365, Perkin Elmer) and a PL spectrophotometer with an Xe lamp (Darsa, PSI Trading), respectively. PLQY values of the QDs were calculated by comparison with standard rhodamine 6G (QY = 95% in ethanol). The crystal phase of the obtained QDs was characterized by X-ray diffractometry (XRD; D/MAX-2500 V, Rigaku). A scanning electron microscope (SEM; JSM-7610F, JEOL, Ltd.) and a transmission electron microscope (TEM; JEM-F200, JEOL, Ltd.) with energy-dispersive X-ray spectroscopy (EDS) were utilized to analyze the size, morphology and crystal structure, and to perform elemental analysis of the obtained red CIS/ZnS, CIS/ZnS/Zr(i-PrO)_4_ and green CGS/ZnS, CGS/ZnS/Zr(i-PrO)_4_ QDs and Al_2_O_3_ encapsulated QD hybrid powders. To analyze the functional groups of ligands in the obtained red CIS/ZnS, CIS/ZnS/Zr(i-PrO)_4_ and green CGS/ZnS, CGS/ZnS/Zr(i-PrO)_4_ QDs, Fourier transform infrared (FT-IR) measurement with an IR spectrophotometer (Nicolet iS50, Thermo Fisher Scientific) was conducted. The EL emission spectra of the fabricated Al_2_O_3_ encapsulated green and red QD hybrid powders single-package down-converted WLEDs were measured using a spectrophotometer (Darsapro-5000, PSI Co. Ltd.).

## Results and Discussion

**Fig. 1:**
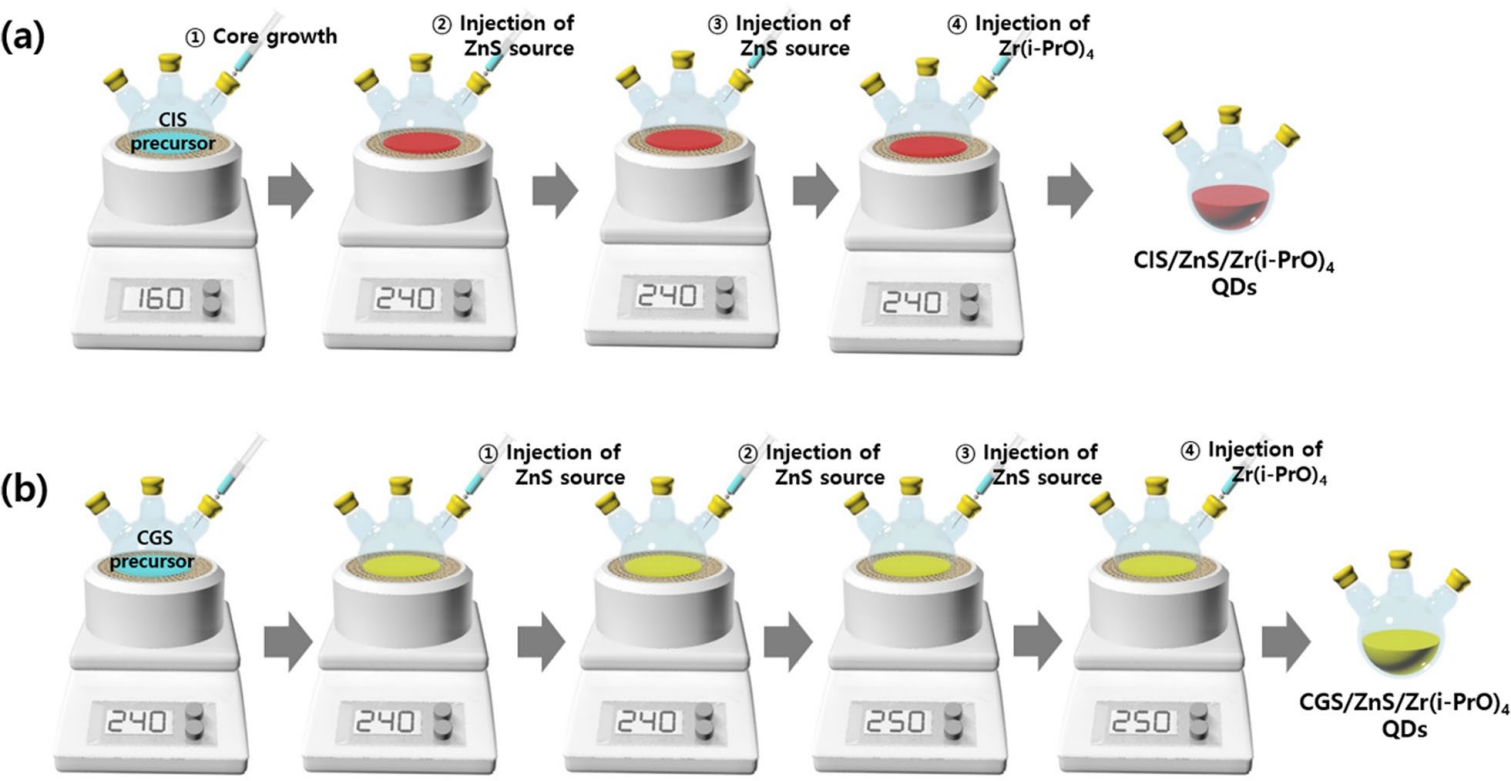
Schematic diagram of both **a** red CIS/ZnS and **b** green CGS/ZnS QDs using multi-step hot-injection method

We synthesized both G-CGS/ZnS and R-CIS/ZnS QDs using a multi-step hot-injection method according to synthetic processes reported in our previous studies (Fig. 1). Both G-CGS/ZnS/Zr(i-PrO)_4_ QDs and R-CIS/ZnS/Zr(i-PrO)_4_ QDs were obtained by in-situ treatment with Zr(i-PrO)_4_ after synthesizing G-CGS/ZnS and R-CIS/ZnS QDs, as previously reported [2, 3].

**Fig. 2:**
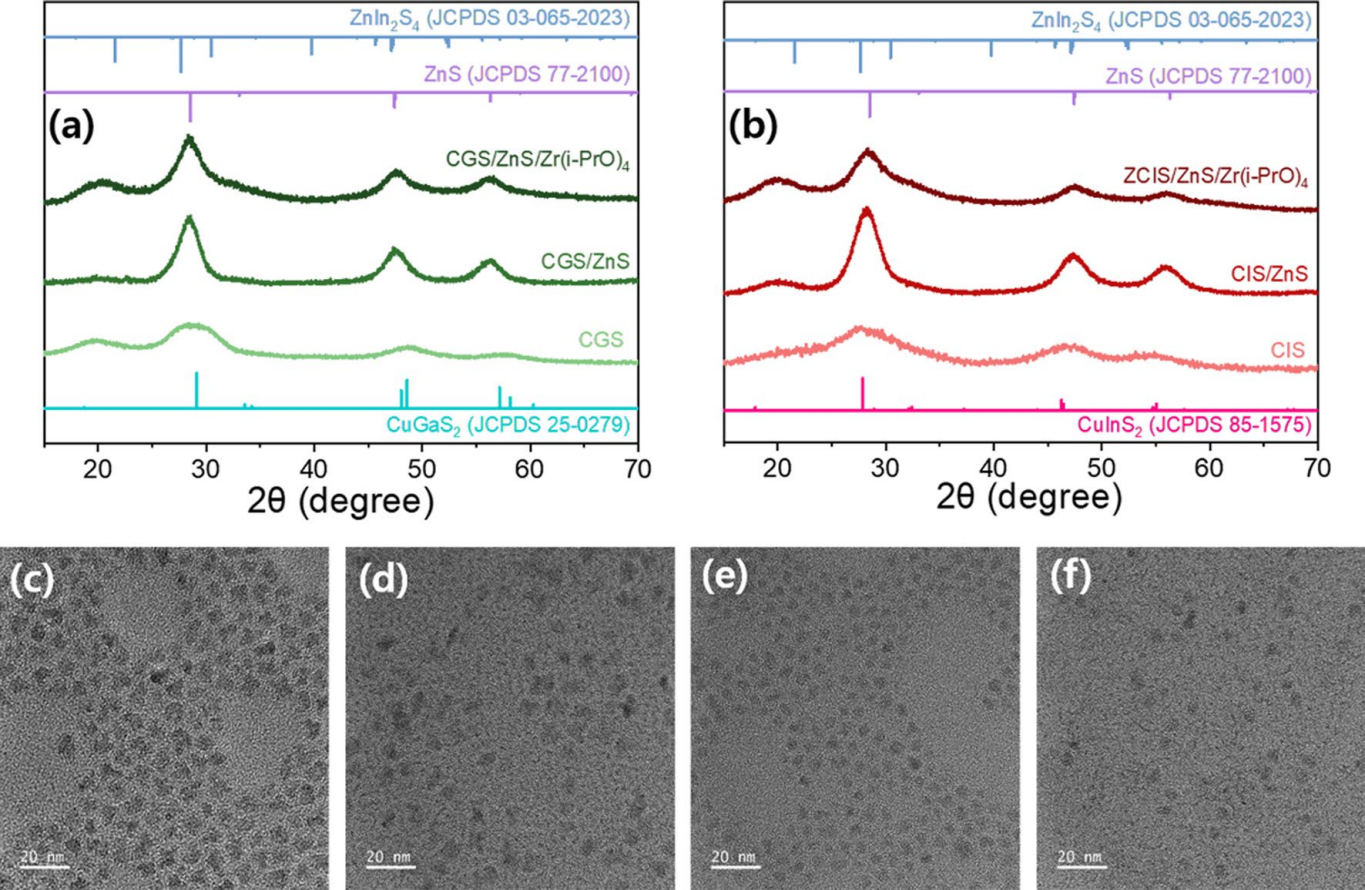
XRD patterns of **a** green QDs of CGS, CGS/ZnS and CGS/ZnS/Zr(i-PrO)_4_ and **b** red QDs of CIS, CIS/ZnS and CIS/ZnS/Zr(i-PrO)_4_. TEM images of green QDs of: **c** CGS/ZnS and **d** CGS/ZnS/Zr(i-PrO)_4_; red QDs of **e** CIS/ZnS and **d** CIS/ZnS/Zr(i-PrO)_4_

The X-ray diffraction (XRD) patterns of the two green QDs of pristine CGS/ZnS and CGS/ZnS/Zr(i-PrO)_4_ and two red QDs of pristine CIS/ZnS and CIS/ZnS/Zr(i-PrO)_4_ are shown in Fig. 2a, b, respectively. The peak shift of the main diffraction peaks of CuGaS_2_ and CuInS_2_ to higher values of 2θ and the merged main peaks of CuGaS_2_ (or CuInS_2_) and ZnS diffraction peaks indicate that alloyed structures were formed in these I-I I–III–VI core/ZnS shell QDs, which matches with the XRD patterns in previous publications [3, 6]. The reduced peak intensity and unchanged peak positions of QDs treated with Zr(i-PrO)_4_ molecules are presumably attributable to the attenuation of diffracted XRD patterns by the Zr(i-PrO)_4_ molecules successfully screened on the ZnS surface. The XRD intensity of larger green QDs is slightly reduced, but that of the smaller red QDs is slightly more reduced by the different screening effects of the Zr(i-PrO)_4_ coating. Figure 2c–f presents transmission electron microscopy (TEM) images of two green and two red pristine and Zr(i-PrO)_4_-decorated QDs. These images indicate that the average sizes of both the G- and R-emitting QDs are little changed after the Zr(i-PrO)_4_ complexing process on the surface of both the G and R I–III–VI QDs (Additional file 1: Fig. S1).

**Fig. 3:**
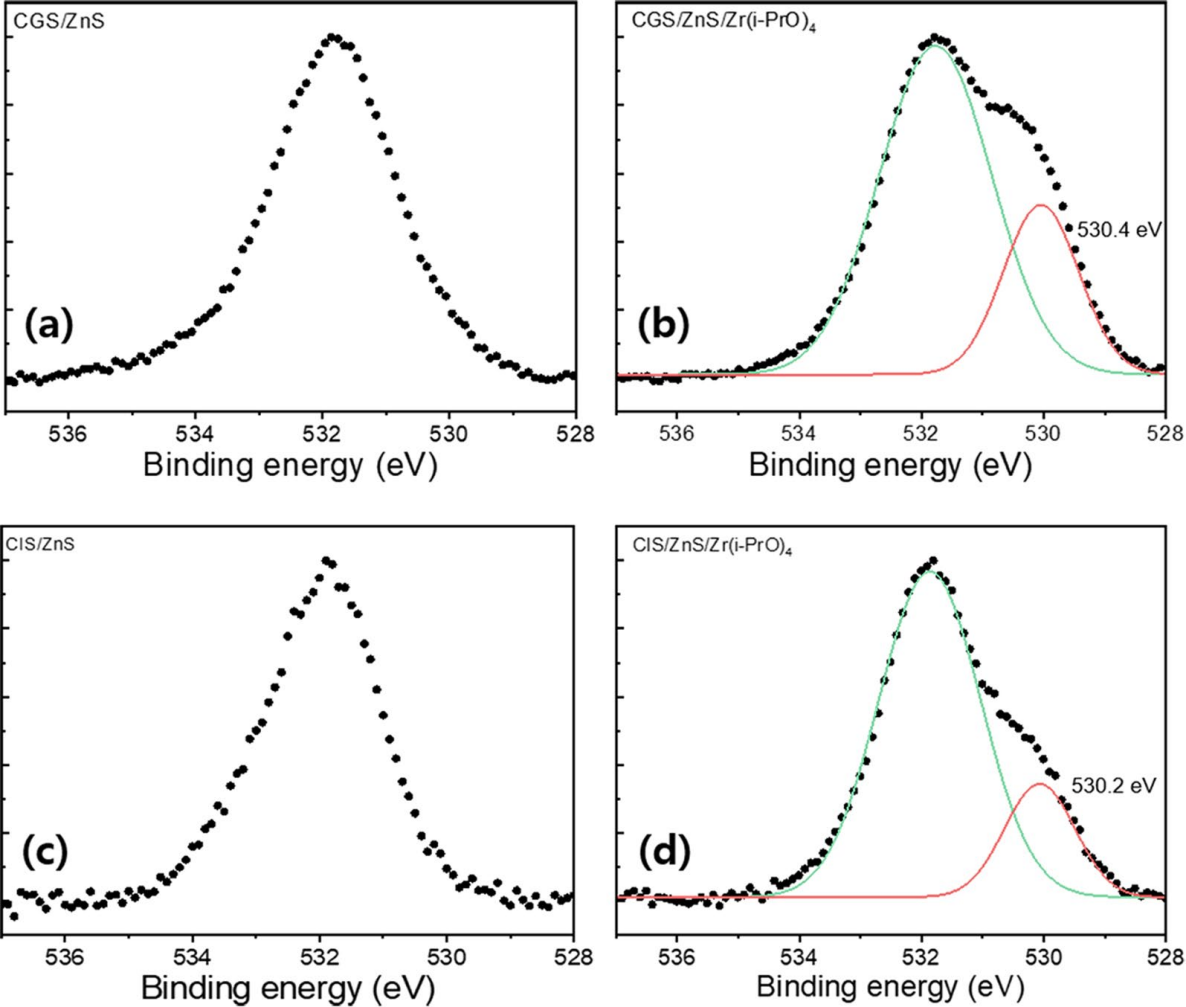
Partial XPS survey results of O component peaks of green **a** CGS/ZnS and **b** CGS/ZnS/Zr(i-PrO)_4_ QDs; and red **c** CIS/ZnS and **d** CIS/ZnS/Zr(i-PrO)_4_ QDs. In the XPS spectra, ZrO_2_ (red line) peak is deconvoluted from the original O spectra (black line)

The TEM images also indicate that two G and two R QDs are suitably grown with single crystal-like nanoparticles. The TEM results imply that the coated Zr(i-PrO)_4_ complexes cannot be transformed into thick ZrO_2_ oxide overcoating via thermal decomposition even at high reaction temperature of 240 ~ 250 °C. XPS analyses were performed to compare two types of QDs, such as pristine and Zr(i-PrO)_4_-decorated G-CGS/ZnS and R-CIS/ZnS QDs. In the high-resolution XPS peaks in Fig. 3, shoulder peaks of the O 1s signals can be distinctly seen after each Zr(i-PrO)_4_ coating process in both G-CGS/ZnS/Zr(i-PrO)_4_ and R-CIS/ZnS/Zr(i-PrO)_4_ QDs due to the Zr-O bonds from metal alkoxide liganded QDs. As reported in previous publications [23, 38], the O 1s peak of unreacted QDs is due to oxygen species from carboxylate QD ligands and atmospheric gaseous oxygen species adsorbed on the QD surface. Otherwise, the O 1s peaks of complexed Zr(i-PrO)_4_-QDs can deconvolute into two sub-spectra. The two peaks of O 1s from the Zr(i-PrO)_4_-coated GR QDs are centered at binding energies of 531.7 and 530.4 eV, respectively [38]. The first 531.7 eV peak of both GR samples is the same as that in the uncoated alloy-core/shell QDs. The second peak is typically an oxygen peak from the Zr–O bond of Zr(i-PrO)_4_. All G and R QD samples show nearly constant peaks of Cu 2d, Ga 2d, Zn 2p, and S 2p_3/2_ for G QDs and Cu 2d, In 3d, and Zn 2p, S 2p_3/2_ for R QDs, respectively [37, 38].

**Fig. 4:**
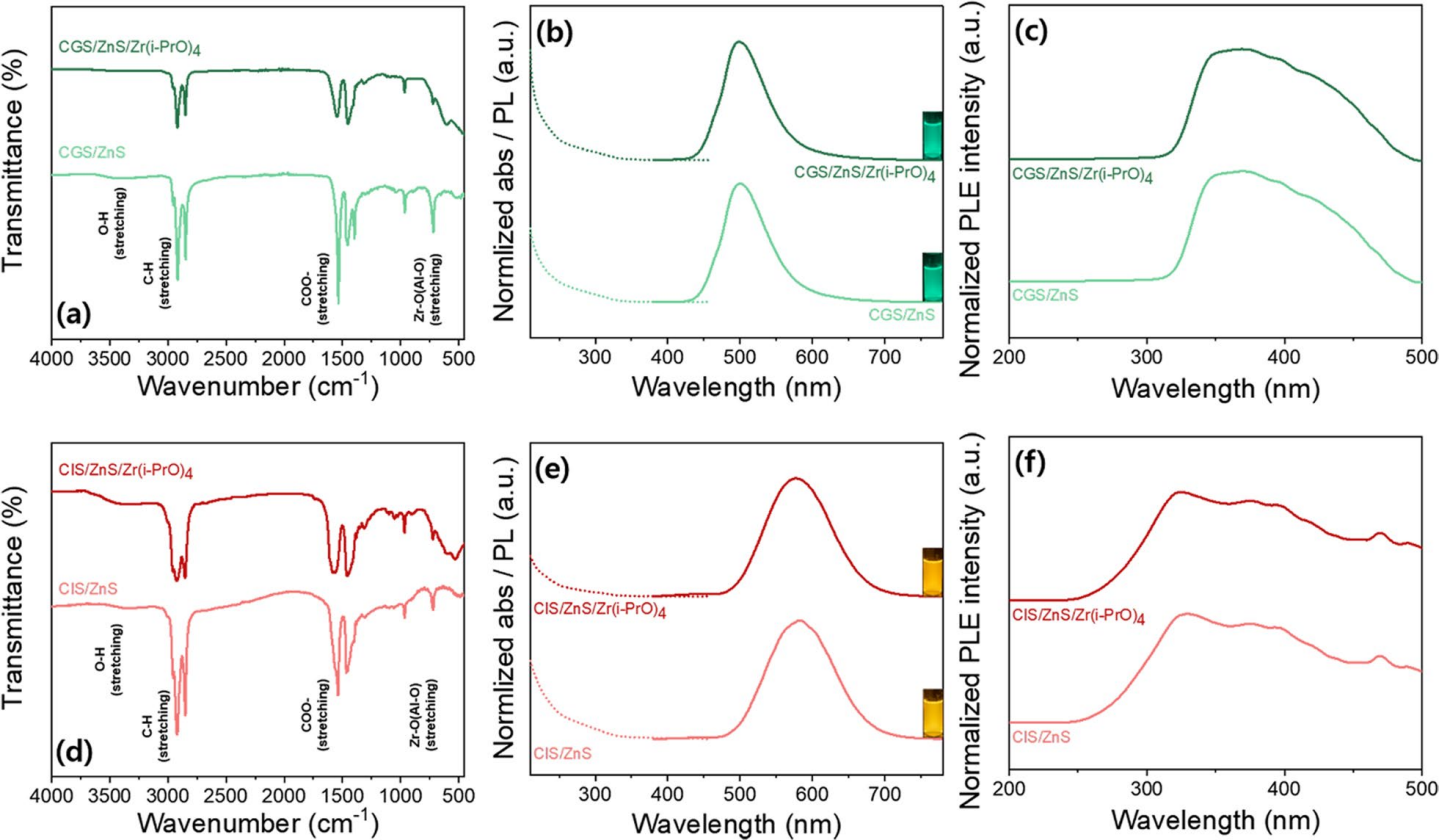
FT-IR spectra of **a** green pristine CGS/ZnS and CGS/ZnS/Zr(i-PrO)_4_ and **d** red pristine CIS/ZnS and CIS/ZnS/Zr(i-PrO)_4_ QD solutions. PL emission spectra, absorbance, and photographs of emitting solutions (inset) of **b** green pristine CGS/ZnS, CGS/ZnS/Zr(i-PrO)_4_ and **e** red pristine CIS/ZnS and CIS/ZnS/Zr(i-PrO)_4_ QD solutions. **c** PLE spectra of green pristine CGS/ZnS, CGS/ZnS/Zr(i-PrO)_4_ and **f** red pristine CIS/ZnS and CIS/ZnS/Zr(i-PrO)_4_ QD solutions

Figure 4a, d shows the FT-IR spectra of the G and R-emitting pristine and Zr(PrO)_4_-decorated QD solutions. The two pristine G and R QD samples commonly show strong bands at 2920, 2850, 1550, and 1450 cm^−1^, which are assigned to –CH_2_, –CH_3_ stretching, COO– antisymmetric stretching mode, and COO– symmetric stretching mode, respectively [23, 37]. These are attributed to aliphatic surface ligands, such as stearate, oleate, and dodecyl and carboxylate groups of fatty acid bonded on the QD surface. As can be seen in the FT-IR spectra, the C–H and COO– stretching modes of the ligand-attached QD solution samples indicate that the ligand signals of the pristine QDs are slightly reduced by the screening effect or the ligand detachment effect of Zr(i-PrO)_4_. The stretching mode of the ligand is still strong, so the ligands are mostly attached to the surface of the QDs, despite the alkoxide coating. The distinct band at 500 cm^−1^ can be associated with the Zr–O stretching band of Zr(i-PrO)_4_ molecules [37]. These results also prove indirectly the suitable complexing on the ZnS surface of QDs with metal alkoxide forms.

Changes in normalized PL, PL excitation (PLE), and absorption spectra of solution forms of pristine QDs and G-CGS/ZnS/Zr(i-PrO)_4_ and R-CIS/ZnS/Zr(i-PrO)_4_ QDs are shown in Fig. 4b, c and Fig. 4e, f. The peak position and full-width at half maximum (FWHM) of the PL emission spectra of G-CGS/ZnS and R-CIS/ZnS QDs are almost unchanged after sequential coating of Zr(i-PrO)_4_ alkoxides. Small differences of the PLE and absorption spectra between the two solution-typed QD samples can be thought to have arisen from the additional absorption of complexing molecules of Zr(i-PrO)_4_ after the Zr(i-PrO)_4_ coating process. Table 1 summarizes the detailed optical properties of the two GR pristine QDs and two Zr(i-PrO)_4_ complexed QDs. As previously reported, the PLQYs of the Zr(i-PrO)_4_ complexed QDs are slightly higher than or similar to those of pristine QDs [38]. As a result, the PLQYs of both resultant G-CGS/ZnS/Zr(i-PrO)_4_ and R-CIS/ZnS/Zr(i-PrO)_4_ QDs reach similar values of ~ 95%. It can be seen that the additional surface passivating process with Zr(PrO)_4_ hardly changed the optical properties, such as peak wavelength, FWHM, and CIE color coordinates of both G-CGS/ZnS and R-CIS/ZnS QDs, as summarized in Table 1. These materials also emit similar bright green and orange-red color before and after Zr(PrO)_4_ complexation, as shown in the insets of Fig. 4b, e. Although it has not been fully established how much Zr(i-PrO)_4_ chemically decorates the surface of QDs, PL spectra results show that these complexing processes preserve the PL properties of pristine GR QD solutions.

**Table 1:** Detailed optical properties of four QD solutions (CGS/ZnS, CGS/ZnS/Zr(i-PrO)_4_, CIS/ZnS and CIS/ZnS/Zr(i-PrO)_4_)

Sample	Color coordinates		Peak wavelength (nm)	FWHM (nm)	PLQY (%)
	CIEx	CIEy			
CGS/ZnS	0.236	0.479	499	71	93
CGS/ZnS/Zr(i-PrO)_4_	0.229	0.472	498	70	95
CIS/ZnS	0.521	0.464	582	108	94
CIS/ZnS/Zr(i-PrO)_4_	0.511	0.470	578	105	95

**Fig. 5:**
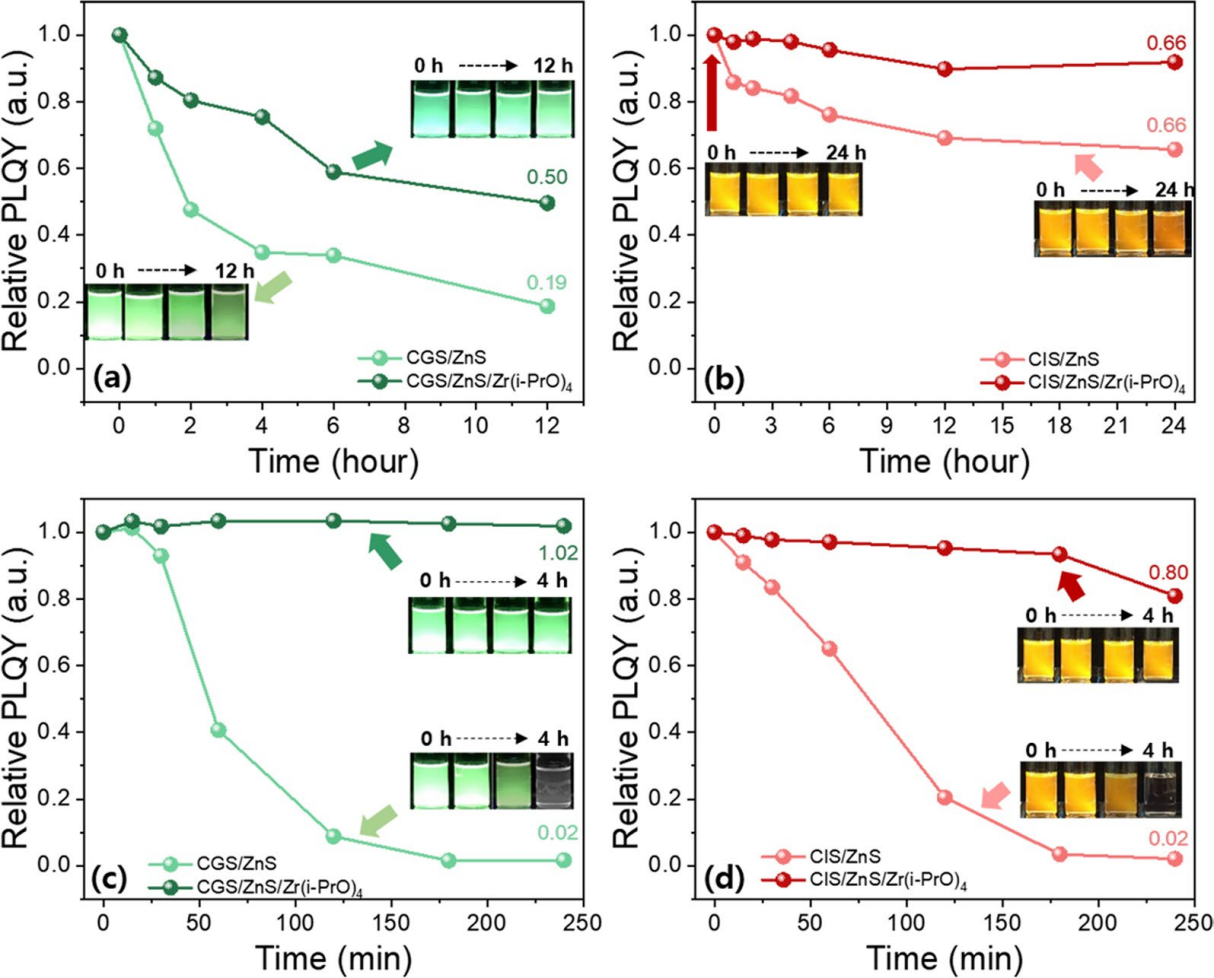
Relative PLQY values from photostability tests of **a** green CGS/ZnS based solutions and **b** red CIS/ZnS based solutions under UV (365 nm) irradiation. Relative PLQY values from thermal stability tests of **c** green CGS/ZnS based solutions and **d** red CZS/ZnS based solutions under 150 °C

Next, to compare the stability of pristine and Zr(i-PrO)_4_-complexed G-CGS/ZnS and R-CIS/ZnS QDs, stability tests were performed in the colloidal solution form. For the solution test, pristine and Zr(i-PrO)_4_-complexed G-CGS/ZnS and R-CIS/ZnS QD colloids were dispersed in ODE and their colloidal solutions were placed under continuous UV (365 nm) irradiation or on a hot plate at 150 °C for prolonged periods of time. As shown in Fig. 5a, b, largely increased stability values of both G and R QDs were observed under UV irradiation, clearly indicating that the passivating effect of the Zr(i-PrO)_4_-complex is strongly effective on the surfaces of both ZnS-shelled G-CGS and R-CIS QDs. Pristine G-CGS/ZnS and R-CIS/ZnS QDs retained values of only 19% and 66% of their original PLQYs after 12 h and 24 h of UV irradiation, respectively.

**Fig. 6:**
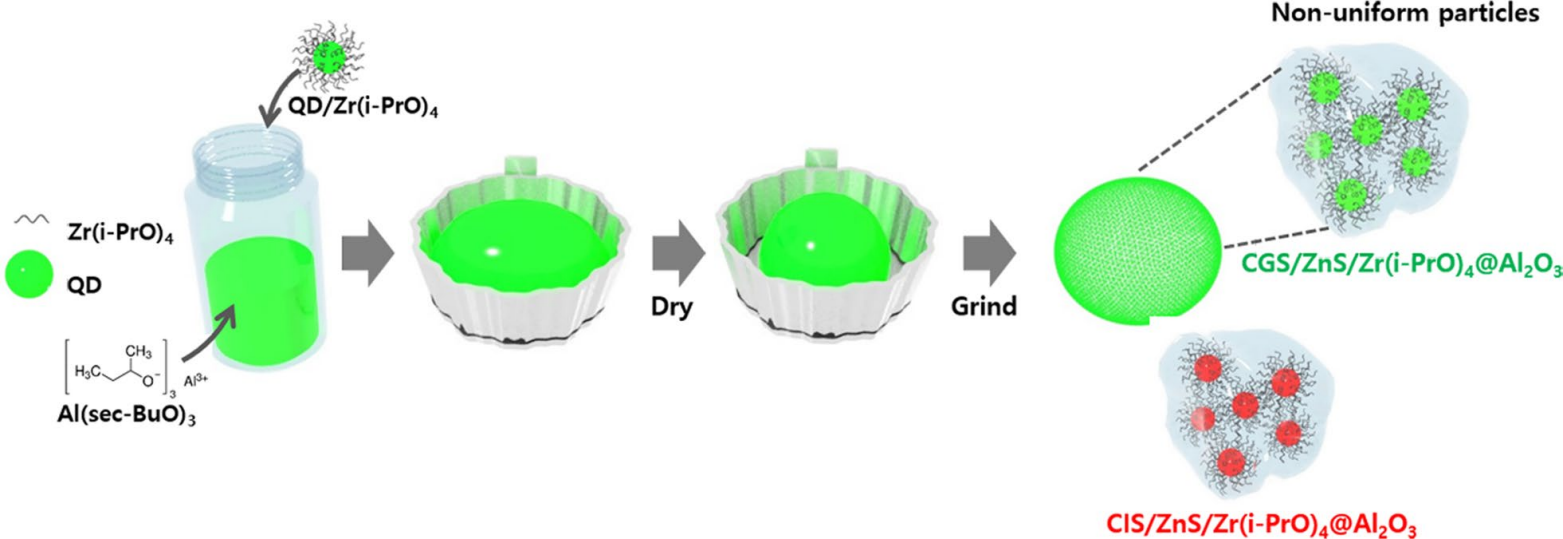
Schematic diagram of ex-situ hydrolysis reaction of Al(sec-BuO)_3_ precursor using purified pristine and Zr(i-PrO)_4_-complexed QD solutions under ambient moisture at room temperature

QD precipitations of both G-CGS/ZnS and R-CIS/ZnS QDs were observed clearly under long-term exposure to UV irradiation, indicating the desorption of liable ligands and flocculation of both G-CGS/ZnS and R-CIS/ZnS QDs by the change in surface hydrophilicity through photochemical reactions. Meanwhile, both Zr(i-PrO)_4_-complexed G-CGS/ZnS and R-CIS/ZnS QDs showed moderate PLQY drops for up to 6 h, retaining ~ 50% of the initial value for green QDs after 12 h and ~ 92% for red QDs after 24 h. The improved photo-stability of Zr(i-PrO)_4_-coated G-CGS/ZnS and R-CIS/ZnS QDs indicates that Zr(i-PrO)_4_-complexed on the surface of QDs suppresses the desorption of ligands from the surfaces of both G-CGS/ZnS and R-CIS/ZnS QDs. The mixture of Zr(i-PrO)_4_ and G-CGS/ZnS and R-CIS/ZnS QDs showed a trend of photo-stability as a function of UV irradiation similar to that of pristine G-CGS/ZnS and R-CIS/ZnS QDs. These trends suggest that non-complexed molecules did not contribute to the surface passivation and that Zr(i-PrO)_4_ was complexed on the surface of QDs; these results match well with the results of the previous report [37]. The thermal stability performances of both Zr(i-PrO)_4_-complexed G-CGS/ZnS and R-CIS/ZnS QDs were assessed by analyzing the degradation of PLQY of both G-CGS/ZnS and R-CIS/ZnS QDs in ODE solution heated to 150 °C. Figure 5c, d shows temporal PLQY drops of pristine and Zr(i-PrO)_4_-complexed G-CGS/ZnS and R-CIS/ZnS QDs under thermal degradation environment. Similar to the temporal degradation of PLQY under UV irradiation, the pristine GR QDs suffered from significant PLQY drops with progress of heating time. PLQY values of both G and R dropped to ~ 2% of the original values of G-CGS/ZnS and R-CIS/ZnS QDs, indicating that the thermal quenching of G-CGS/ZnS and R-CIS/ZnS QDs is due to oxidation and surface desorption of ligands. In contrast to the results of pristine QDs, ~ 102% and ~ 81% of initial PLQY values of both Zr(PrO)_4_-complexed G-CGS/ZnS and R-CIS/ZnS QDs were maintained at 150 °C for the same period time. The slight shift of PL peaks of both pristine GR QDs explains the weakening phenomena of quantum confinement through the desorption of ligands and/or surface oxidation. Both temporal UV irradiation and heating test clearly suggested that the coating process of the Zr(i-PrO)_4_-complex on the surface of both G-CGS/ZnS and R-CIS/ZnS QDs is an effective way to form a second passivation layer by reducing the photooxidation and desorption of ligands. To study the additional protection effect of the second Zr(i-PrO)_4_ passivation interlayer during the oxide coating process, QD-embedded Al_2_O_3_ hybrid powders were synthesized by ex-situ hydrolysis reaction of Al(sec-BuO)_3_ precursor using purified pristine and Zr(i-PrO)_4_-complexed QD solutions under ambient moisture at room temperature, as shown in Fig. 6.

**Fig. 7:**
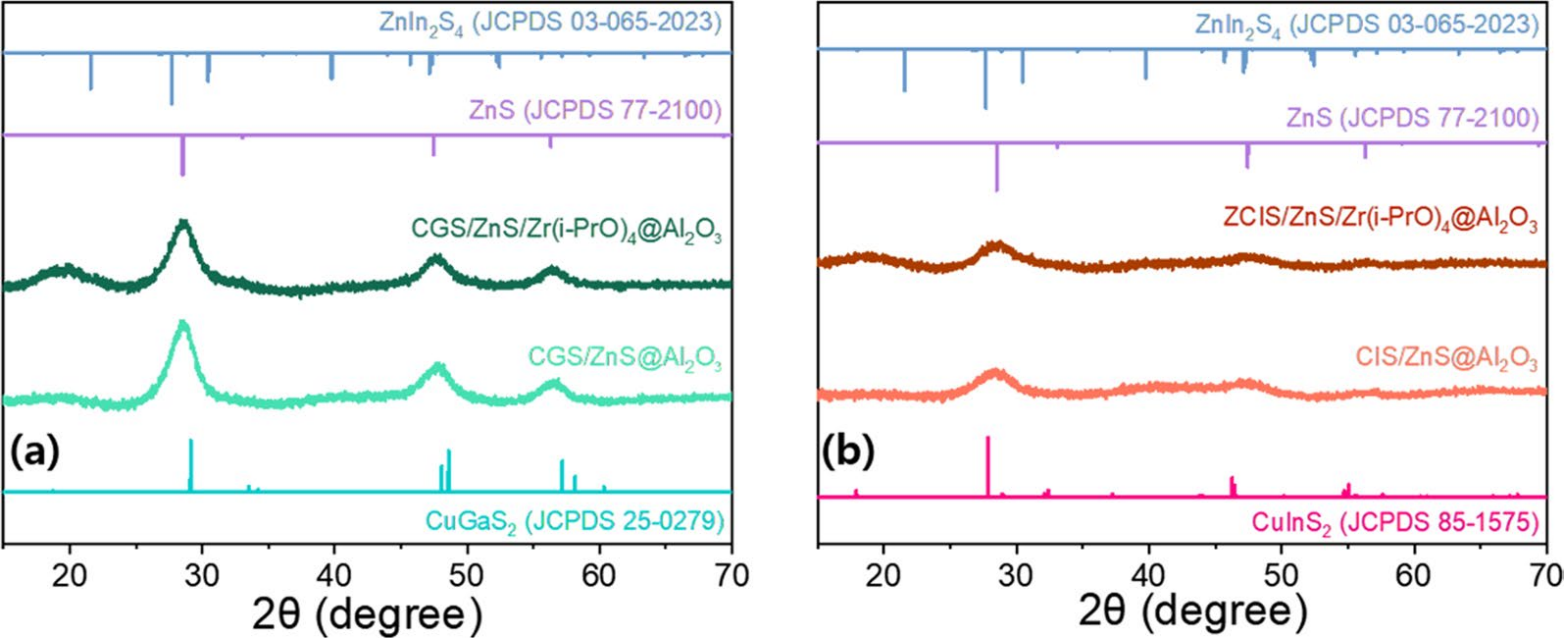
XRD patterns of final Al_2_O_3_ encapsulated **a** green CGS/ZnS based and **b** red CIS/ZnS based powder QDs

Figure 7 also shows that the XRD patterns of the final Al_2_O_3_ encapsulated G-CGS/ZnS and R-CIS/ZnS powder QDs remain in similar positions but their intensities are significantly reduced by the screening of Al_2_O_3_ coating materials. Moreover, the XRD peaks of Al_2_O_3_- and Al_2_O_3_/Zr(i-PrO)_4_-coated G-CGS/ZnS and R-CIS/ZnS QDs are combined with an amorphous XRD hump of the Al_2_O_3_ matrix. Despite the hydrolysis reaction of Al(sec-BuO)_3_ and the induced hydrolysis and complexation of Zr(i-PrO)_4_ at room temperature, the crystallinity of the QDs is maintained.

TEM images of the final Al_2_O_3_- and Al_2_O_3_/Zr(i-PrO)_4_-coated G-CGS/ZnS and R-CIS/ZnS QDs show that the QDs are well dispersed in the amorphous Al_2_O_3_ matrix after the quick hydrolysis reaction of Al(sec-BuO)_3_ with ambient moisture and partially-induced hydrolysis reaction of Zr(i-PrO)_4_ (Additional file 1: Fig. S2a–d). As shown in the SEM pictures and EDS data of Additional file 1: Fig. S2e–h and Fig. 3 ~ S4, the uniformly scattered Zr ions and uniformly distributed Al ions over the entire surface of the irregular-shaped micro-powders indicate that the I–III–VI QDs are coated with double alkoxide/oxides as doubly-passivated/encapsulated layer. Also, uniformly scattered Cu, Ga, In, Zn, and S ions indicate that the QDs are separately and uniformly embedded in the Al_2_O_3_ matrix, though their intensities are relatively low due to the screening effect of the Al_2_O_3_ matrix. As shown in the XPS data in Additional file 1: Fig. S5, the O 1s peaks from Al_2_O_3_-coated G-CGS/ZnS and R-CIS/ZnS QDs and Al_2_O_3_/Zr(i-PrO)_4_-coated G-CGS/ZnS and R-CIS/ZnS QDs show two and three sub-spectra. However, in contrast to expectations, only one strong O 1s peak is shown after the Al_2_O_3_ encapsulating process. This means that the signals from the oxygen peaks of both pristine and Zr(i-PrO)_4_-coated G-CGS/ZnS and R-CIS/ZnS QDs are screened by the thick encapsulant matrix of the Al_2_O_3_ powder matrix and then disappear. All XPS spectra indicate that the binding energy of the restrictive photoelectrons of each element from the QDs remains nearly unchanged even after Al_2_O_3_ encapsulant coating; however, due to the screening effect of additional metal alkoxide and oxide layers, these peak intensities somewhat decrease with increased number of sequential coatings of Zr(i-PrO)_4_ and Al_2_O_3_. FT-IR measurements were performed on Al_2_O_3_- and Al_2_O_3_/Zr(i-PrO)_4_-coated G-CGS/ZnS/ and R-CIS/ZnS QDs to further support the results of XPS analysis.

**Fig. 8:**
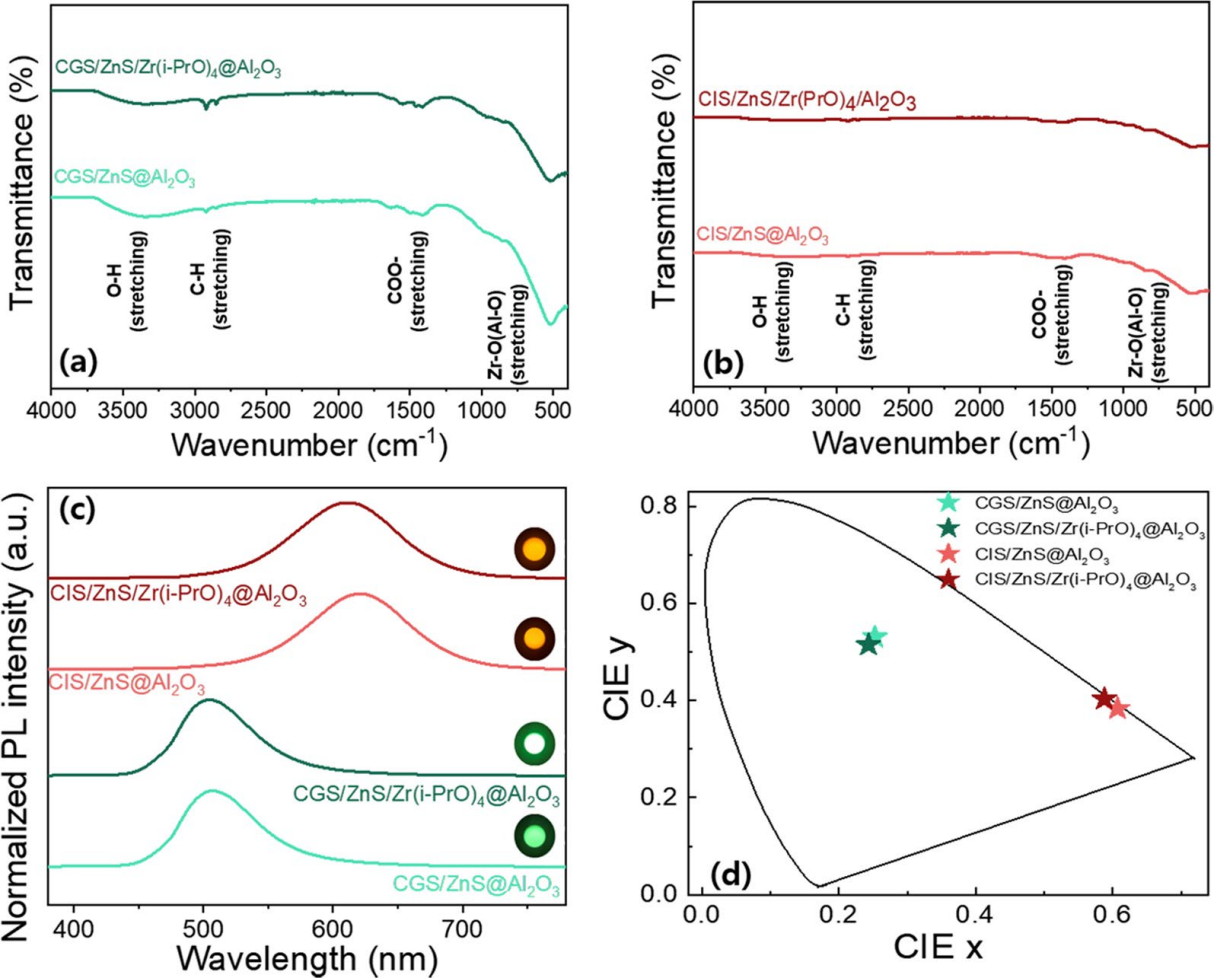
FT-IR spectra of Al_2_O_3_ encapsulated **a** CGS/ZnS based powder QDs and **b** CIS/ZnS based powder QDs. **c** PL emission spectra and photographs of emitting powder QDs (inset) and **d** CIE color coordinates of Al_2_O_3_ encapsulated of CGS/ZnS based powder QDs and CIS/ZnS based powder QDs

Figure 8a, b, indicates that all –CH_2_, –CH_3_, COO– antisymmetric, and COO– symmetric stretching modes are significantly reduced by the screening effect of Al_2_O_3_ encapsulant. The increased IR signals indicate that –CH_2_ and –CH_3_ stretchings of the Al_2_O_3_/Zr(i-PrO)_4_-coated G-CGS/ZnS and R-CIS/ZnS QD powders are stronger than those of the Al_2_O_3_-coated G-CGS/ZnS and R-CIS/ZnS QD powders, as shown Fig. 8a, b. This means that the passivation effect of the Zr(i-PrO)_4_-complex keeps more ligands attached to the surface of the ZnS shell during the Al_2_O_3_ encapsulating process of the fast hydrolysis precursor of Al(sec-BuO)_3_. As mentioned above, the distinct band at 500 cm^−1^ is associated with Zr-O stretching of the Zr(i-PrO)_4_ complex [32]. However, these figures also show that FT-IR peaks from Al_2_O_3_-, Al_2_O_3_/Zr(i-PrO)_4_-coated G-CGS/ZnS, and R-CIS/ZnS QDs consist of two bands from Al-O stretching (and/or Zr-O stretching), as well as common aliphatic and carboxy stretching bands. The different subpeak positions and shapes of the Al-O stretching bands between Al_2_O_3_-coated QDs and Al_2_O_3_/Zr(i-PrO)_4_-coated QDs support the idea that the Al-O stretching band can screen the Zr-O stretching bands in Al_2_O_3_/Zr(i-PrO)_4_-coated QD powders. Therefore, even if the complexing molecules of Zr(i-PrO)_4_-coated QDs can be partially transformed by induced hydrolysis into oxide-layer encapsulated QDs, it can be assumed that the final encapsulated/passivated QDs can be expressed as Al_2_O_3_/Zr(i-PrO)_4_-coated G-CGS/ZnS and R-CIS/ZnS QDs during the fast and strong hydrolysis reaction of the reactive Al(sec-BuO)_3_ precursor. To compare the effects of inner Zr(i-PrO)_4_ coating produced via complexation or partially-induced hydrolysis on PLQY values and stability of G-CGS/ZnS@Al_2_O_3_ and R-CIS/ZnS QDs@Al_2_O_3_ solid powders, the optical properties of both G-CGS/ZnS@Al_2_O_3_ and R-CIS/ZnS QDs@ Al_2_O_3_ and G-CGS/ZnS/Zr(i-PrO)_4_@Al_2_O_3_ and R-CIS/ZnS/Zr(i-PrO)_4_@Al_2_O_3_ QD hybrid powders were analyzed. As shown in Figs. 4 and 8, after producing powder QDs by drying and Al_2_O_3_ encapsulating process, the peak positions of PL spectra of the G-CGS/ZnS and R-CIS/ZnS QD samples are slightly or significantly red-shifted from solution to powder. This red-shift is mainly attributed to greater agglomeration of QDs during the solidification process following either the drying or encapsulating process of Al_2_O_3_. The PLEs of the G-CGS/ZnS and R-CIS/ZnS QD solid powders complexed by Zr(i-PrO)_4_ are almost identical of those of the pristine G-CGS/ZnS and R-CIS/ZnS QD solid powders. However, the PLEs of the Al_2_O_3_- and Al_2_O_3_/Zr(i-PrO)_4_-coated G-CGS/ZnS and R-CIS/ZnS QD samples are red-shifted from those of the QD solutions due to the agglomeration or increased size of QDs, resulting in an increased excitation intensity of blue light from a blue LED (Fig. 4 and Additional file 1: Fig. S6). The resulting Al_2_O_3_/Zr(i-PrO)_4_-coated G-CGS/ZnS and R-CIS/ZnS QDs become more appropriate for use as color converters to convert blue to green and blue to red. As shown in Additional file 1: Fig. S6, like the small change of Zr(i-PrO)_4_ complexed QDs in solutions, a slight alteration of the absorption of the Zr alkoxide complexed QDs is observed in the last Al_2_O_3_ powders. Strong absorption peaks below 400 nm for all powder samples suggest that the surfaces of the QDs are encapsulated with an oxide layer of Al_2_O_3_ or Al_2_O_3_/Zr(i-PrO)_4_.

The outer Al_2_O_3_ coatings convert QD solution forms into ready-to-use QD powder forms. However, the Zr(i-PrO)_4_ interlayer slightly blueshifts the peak wavelengths of both Al_2_O_3_-coated G-CGS/ZnS and R-CIS/ZnS QDs owing to the increase in distances among QDs (Fig. 8c, d, Table 2). In Table 2, it can be clearly seen that PLQYs improved slightly from 48.7 to 53.7% for green QDs and from 44.1 to 52.2% for red QDs after insertion of Zr(i-PrO)_4_ interlayer between QD surfaces and Al_2_O_3_ outermost protective layer. The increase in PLQY can also suggest that the Al_2_O_3_ single layer coating likely plays a synergistic role in passivating and restoring QD defects formed by the Zr(i-PrO)_4_ interlayer. However, for application to WLEDs, it is necessary to evaluate the effect of the Zr(i-PrO)_4_ interlayer on the environmental stability of the outermost Al_2_O_3_ protective layer of the G-CGS/ZnS and R-CIS/ZnS QDs against UV, temperature, and moisture.

**Table 2:** Detailed optical properties of Al_2_O_3_ encapsulated CGS/ZnS based powder QDs and CIS/ZnS based powder QDs

Sample	Color coordinates		Peak wavelength (nm)	FWHM (nm)	PLQY (%)
	CIEx	CIEy			
CGS/ZnS@Al_2_O_3_	0.253	0.531	507	68	48.7
CGS/ZnS/Zr(i-PrO)_4_@Al_2_O_3_	0.244	0.515	504	66	53.7
CIS/ZnS@Al_2_O_3_	0.607	0.384	621	90	44.1
CIS/ZnS/Zr(i-PrO)_4_@Al_2_O_3_	0.588	0.403	611	93	52.2

**Fig. 9:**
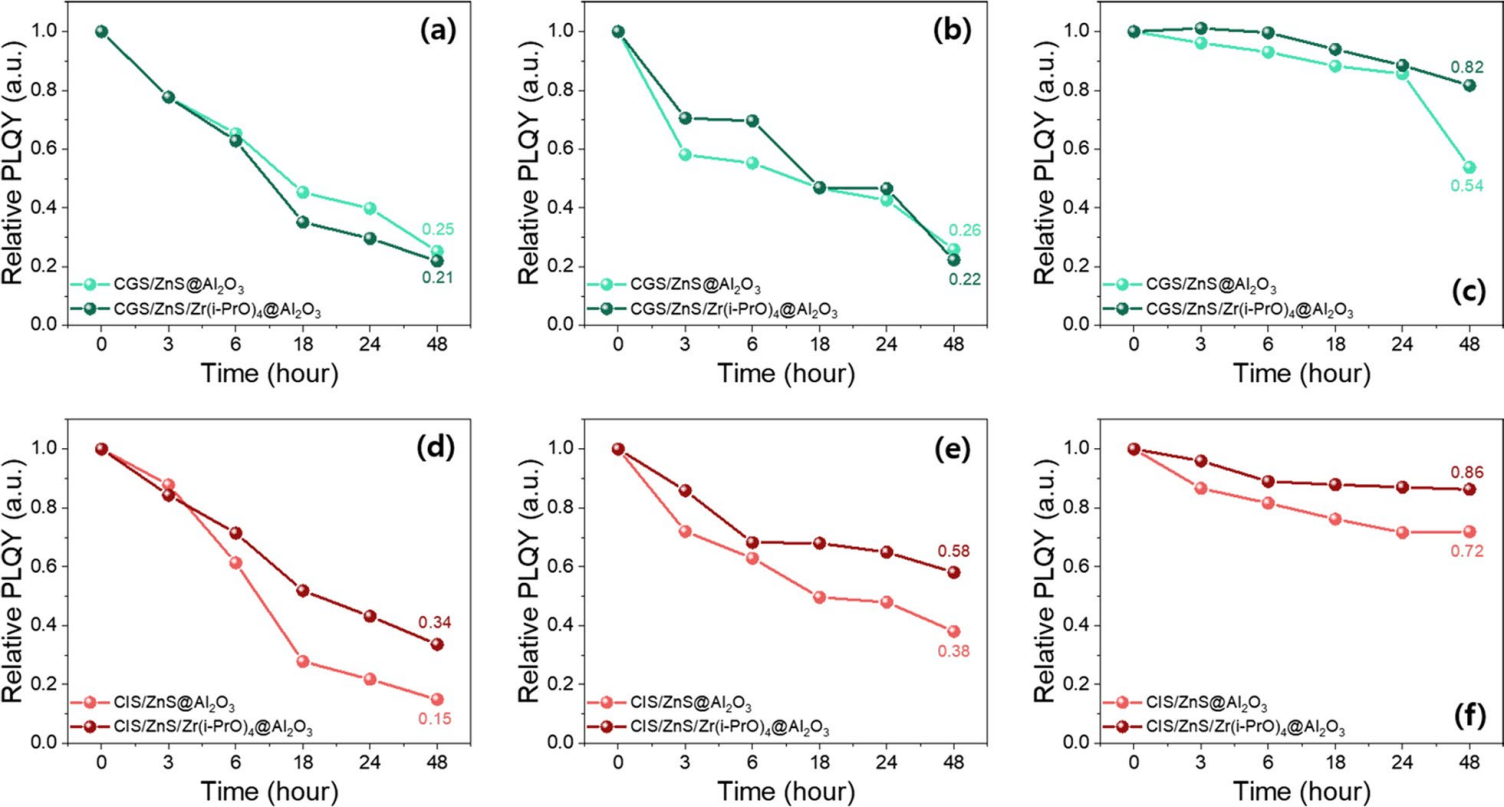
**a** Photograph (under UV irradiation) and **b** thermal stability (120 °C heating condition) and **c** moisture resistance (under 85H/85T) of green Al_2_O_3_ encapsulated CGS/ZnS based QD hybrid powders. and **d** photograph and **e** thermal stability and **f** moisture resistance of red Al_2_O_3_ encapsulated CIS/ZnS based QD hybrid powders

**Fig. 10:**
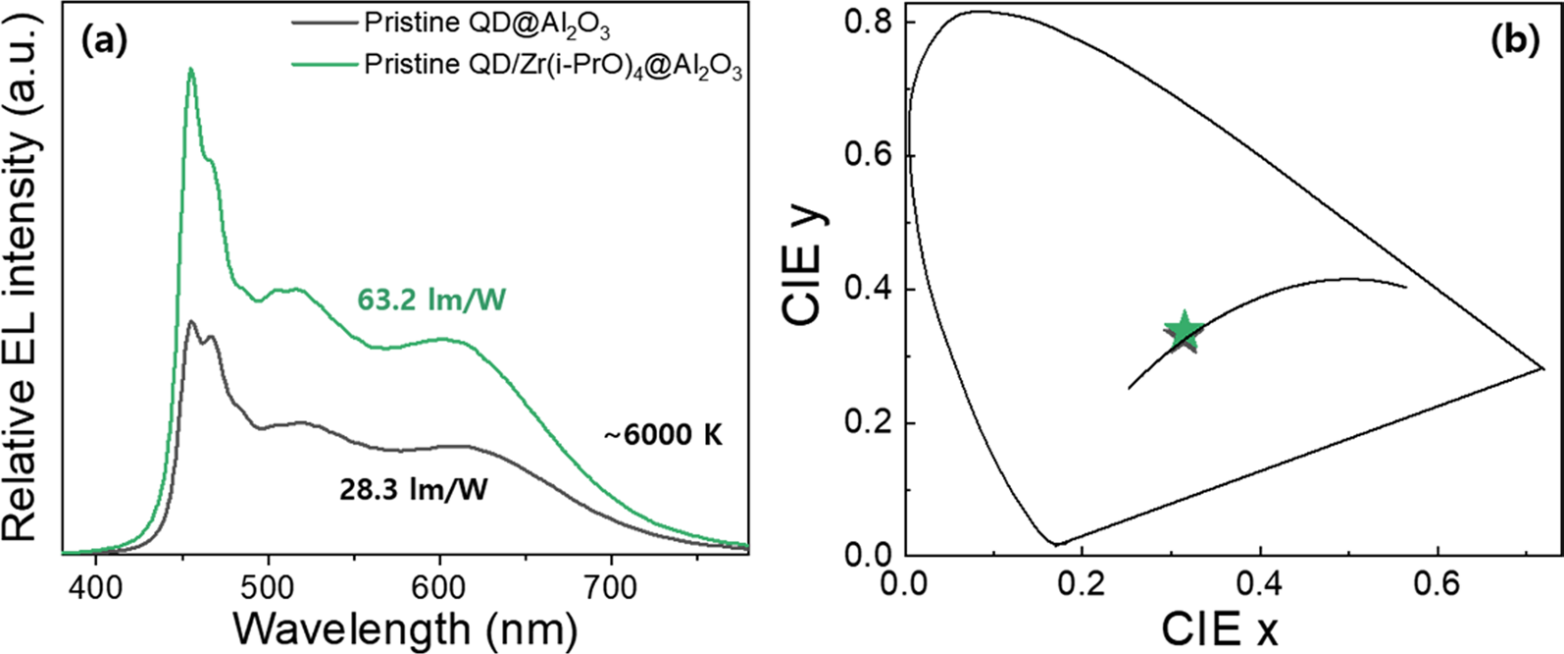
**a** EL spectra and **b** CIE color coordinates of two correlated color temperatures (CCTs) of fabricated pristine QD@Al_2_O_3_ and pristine QD/Zr(i-PrO)_4_@Al_2_O_3_ QD hybrid powders in DC-WLED package

Here, to compare the stability of the Al_2_O_3_-coated and Al_2_O_3_/Zr(i-PrO)_4_-coated G-CGS/ZnS and R-CIS/ZnS QDs, stability tests were performed on the solid powder form according to the phase of QDs obtained from fast hydrolysis reaction. As shown in Fig. 9, the temporal photo-stability, thermal-stability, and moisture-resistance of the solid-state forms of Al_2_O_3_-coated G-CGS/ZnS and R-CIS/ZnS QDs and Al_2_O_3_/Zr(i-PrO)_4_-coated G-CGS/ZnS and R-CIS/ZnS QDs were tested under 365 nm UV irradiation, 120 °C heating condition, and 85% humidity/85 °C temperature (85H/85T) for a prolonged period. Because they are obtained as colloidal solutions, which cannot be transformed into solid forms, it is not reasonable to directly compare the stabilities of pristine and Zr(i-PrO)_4_-complexed G-CGS/ZnS and R-CIS/ZnS QD solutions with those of solid forms of oxide-coated QDs. As shown in Fig. 9, both Al_2_O_3_-coated G-CGS/ZnS and R-CIS/ZnS QDs experienced moderate PLQY drops during UV irradiation, heat treatment, and moisture treatment of up to 50 h, retaining 25, 26, and 54% of original PLQYs of G QDs and 15, 38, and 72% of original PLQYs of R QDs. In the presence of a Zr(i-PrO)_4_ interlayer, PLQY values of Al_2_O_3_-coated G-CGS/ZnS QD powders drop to 21, 22, and 82% after 50 h under heat, temperature, and moisture treatment, respectively. In the G-CGS/ZnS QDs, the stability is improved only under the 85H/85T condition; the interlayer has little effect on the stability under UV irradiation and heating conditions. On the other hand, in the case of R-CIS/ZnS QDs, the PLQY of Al_2_O_3_/Zr(i-PrO)_4_-coated R-CIS/ZnS QD powders drops to 34, 58, and 86% after 50 h treatment of UV, heat, and moisture conditions, respectively. These graphs show that the stabilities of Al_2_O_3_/Zr(i-PrO)_4_-coated QD powders are improved by the presence of a Zr(i-PrO)_4_ interlayer under all UV, heat, and 85H/85T conditions. Although G-CGS/ZnS@Al_2_O_3_ QD powders are not improved under all conditions, the improvement of R-CIS/ZnS@Al_2_O_3_ QD powders under all conditions and G-CGS/ZnS Al_2_O_3_ QD hybrid powders under 85H/85T condition clearly show that an Zr(i-PrO)_4_ interlayer is needed for further encapsulation process. The coating effect of the Zr(i-PrO)_4_ interlayer of the Al_2_O_3_/Zr(i-PrO)_4_ layer on the stability of G-CGS/ZnS is not so small compared with the encapsulating effect of the outermost Al_2_O_3_ coating layer. The significant enhancements in photo-stability, thermal-stability, and moisture-stability of the Al_2_O_3_/Zr(i-PrO)_4_-coated R-CIS/ZnS samples clearly confirm that double Al_2_O_3_/Zr(i-PrO)_4_-coatings are effective in passivating and protecting QD surfaces, as well as in forming solid state forms. To compare the effects of the Zr(i-PrO)_4_ interlayer on the G-CGS/ZnS@Al_2_O_3_ and R-CIS/ZnS@Al_2_O_3_ QD powders in the DC-WLED package, two different WLED packages were fabricated by depositing Al_2_O_3_-coated G-CGS/ZnS and R-CIS/ZnS and Al_2_O_3_/Zr(i-PrO)_4_-coated G-CGS/ZnS and R-CIS/ZnS QD powder pastes on blue LED cup-typed dies with two correlated color temperatures (CCTs) of 6500 K. At a current of 60 mA, the LE values of both Al_2_O_3_-coated QDs and Al_2_O_3_/Zr(i-PrO)_4_-coated QD-based WLEDs were found to be 40.44 and 63.2 lm/W in 6500 K. Regardless of the LE value, all four white LEDs showed high CRI values of 91 or 92 owing to the large FWHM (over 80 nm) of the G-CGS/ZnS and R-CIS/ZnS QDs. The comparison of LE values of WLEDs provides a direct comparison of the stability of the QD powders during fabrication of WLEDs filling the two different QD powder pastes in the LED cup-typed die. Figure 10 indicates that the LEs of the Al_2_O_3_/Zr(i-PrO)_4_-coated I–III–VI QD-based WLEDs are improved 1.56 times compared to pristine Al_2_O_3_-coated G-CGS/ZnS and R-CIS/ZnS QD-based WLEDs, respectively.

These increases occurred because the Zr(i-PrO)_4_ interlayer mitigates the degradation of both Al_2_O_3_-coated G-CGS/ZnS and R-CIS/ZnS QD powders during fabrication of DC-WLEDs. Based on the improved optical properties, it is clear that the G-CGS/ZnS/Zr(i-PrO)_4_@Al_2_O_3_ and R-CIS/ZnS/Zr(i-PrO)_4_@Al_2_O_3_ QD powders, synthesized by fast hydrolysis of Al(sec-BuO)_3_ precursor and partially-induced hydrolysis of (Zr(i-PrO)_4_) precursor, are suitable for application to DC-WLEDs with high CRI. Although the LEs of WLEDs incorporating were greatly improved by insertion of Zr(i-PrO)_4_ interlayer, these LE values are still significantly smaller than those of commercially available DC-WLEDs, which use inorganic phosphors. In order to commercialize WLEDs using QD-embedded Al_2_O_3_ powders, additional protection materials and technology are required to minimize the degradation of optical properties of QDs during the harsh reaction needed to produce the QD-embedded oxide powders. Here, it is clearly seen that the introduction of a Zr(i-PrO)_4_ passivation layer and interlayer improve the environmental stability of the eco-friendly I–III–VI QDs itself, and mitigates the further degradation of the I–III–VI QDs during additional inorganic layer coating and fabrication of WLED device.

## Conclusion

The Zr(i-PrO)_4_ complex was decorated on the surfaces of the G-CGS/ZnS and R-CIS/ZnS QDs to suppress ligand loss stemming from the secondary passivation layer. The PLQYs of the G-CGS/ZnS/Zr(i-PrO)_4_ and R-CIS/ZnS Zr(i-PrO)_4_ QD solutions reached similar values of ~ 95%. Their photostability and thermal stability were improved via surface oxidation, suppression of ligand loss, and Zr(i-PrO)_4_ complex decoration-assisted QDs agglomeration. In addition, the Zr(i-PrO)_4_ complex interlayer improves the optical properties of G-CGS/ZnS/Zr(i-PrO)_4_@Al_2_O_3_ and R-CIS/ZnS/Zr(i-PrO)_4_@Al_2_O_3_ QD hybrid powders during synthesis by fast hydrolysis of mixture solution of highly reactive Al(sec-BuO)_3_ precursor and Zr(i-PrO)_4_-decorated G-CGS/ZnS and R-CIS/ZnS QDs. Therefore, the PLQYs of the G-CGS/ZnS/Zr(i-PrO)_4_@Al_2_O_3_ and R-CIS/ZnS/Zr(i-PrO)_4_@Al_2_O_3_ QD hybrid powders reached 53.7 and 52.2% respectively, and material photostability and thermal stability also improved by surface oxidation, suppression of ligand loss, and Zr(i-PrO)_4_ complex decoration-assisted QD agglomeration. The effect of Zr(i-PrO)_4_ secondary passivation on the stability and PLQY values of the I–III–VI QDs and QD-embedded Al_2_O_3_ hybrid powders was studied by analyzing XRD, TEM, FT-IR, and XPS results to determine optical properties after coating of Zr(i-PrO)_4_ on QDs and encapsulating Zr(i-PrO)_4_-coated QDs with Al_2_O_3_ matrix. Finally, single WLED packages were fabricated using two sets of pristine GR QD@Al_2_O_3_ and GR Zr(i-PrO)_4_-QD@Al_2_O_3_ QD hybrid powders. The LEs of the two WLEDs implemented with G-CGS/ZnS/Zr(i-PrO)_4_@Al_2_O_3_ and R-CIS/ZnS/Zr(i-PrO)_4_@Al_2_O_3_ QD hybrid powders improved 2.25 and 2.40 times compared to those of cool and warm color WLEDs implemented with G-CGS/ZnS@Al_2_O_3_ and R-CIS/ZnS@Al_2_O_3_ QD hybrid powders, respectively. Although the currently developed I–III–VI /Zr(i-PrO)_4_@Al_2_O_3_ QD hybrid powders cannot compete with commercialized inorganic phosphor powders, the introduction of a second passivation layer and interlayer of Zr(i-PrO)_4_ provides a simple synthetic process to produce easy-to-use QD powders with improved optical properties for application to eco-friendly I–III–VI QD-based lighting and display devices.

## Supplementary Information

**Additional file 1**. Supplementary figures.

## Data Availability

All data generated or analyzed during this study are included in this published article.
