# Supplementary material for: Passivation and Interlayer Effect of Zr(i-PrO)4 on Green CuGaS2/ZnS/Zr(i-PrO)4@Al2O3 and Red CuInS2/ZnS/Zr(i-PrO)4@Al2O3 QD Hybrid Powders
Source: Nanoscale Res Lett. 2022 Nov 7;17:106. doi: 10.1186/s11671-022-03741-0 (PMC9640527; doi:10.1186/s11671-022-03741-0)
Supplement: Supplementary file 1 — Additional file 1. Supplementary figures. [file 11671_2022_3741_MOESM1_ESM.docx]

**SUPPLEMENTARY INFORMATION**

**Passivation and Interlayer Effect of Zr(i-PrO)_4_ on Green CuGaS_2_/ZnS/Zr(i-PrO)_4_@Al_2_O_3_ and Red CuInS_2_/ZnS/Zr(i-PrO)_4_@ Al_2_O_3_ QD Hybrid Powders**

Minji Ko^1^, Soyeon Yoon^1^, Yun Jae Eo^1^, Yoonji Yang^1^, Keyong Nam Lee^1^ and Young Rag Do*^1^

^1^Department of Chemistry, Kookmin University, Seoul 02707, Republic of Korea

*corresponding author email: [yrdo@kookmin.ac.kr](mailto:yrdo@kookmin.ac.kr)


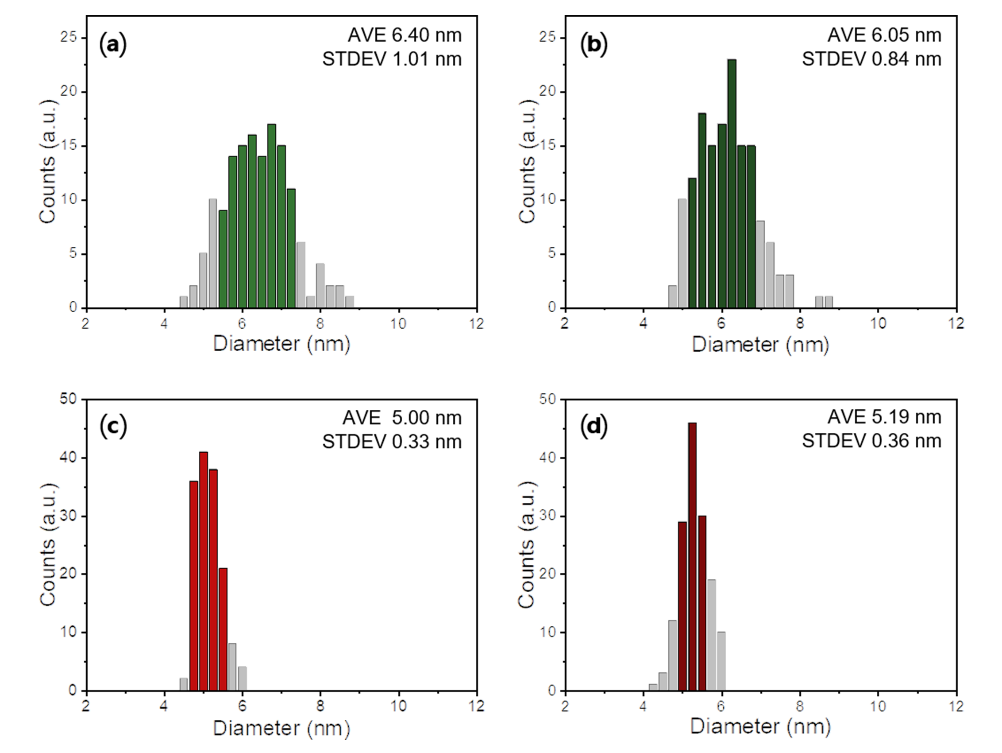


**Fig. S1.** The size distribution of green QDs of (a) CGS/ZnS, (b) CGS/ZnS/Zr(i-PrO)_4_ and red QDs of (c) CIS/ZnS, (d) CIS/ZnS/Zr(i-PrO)_4_.


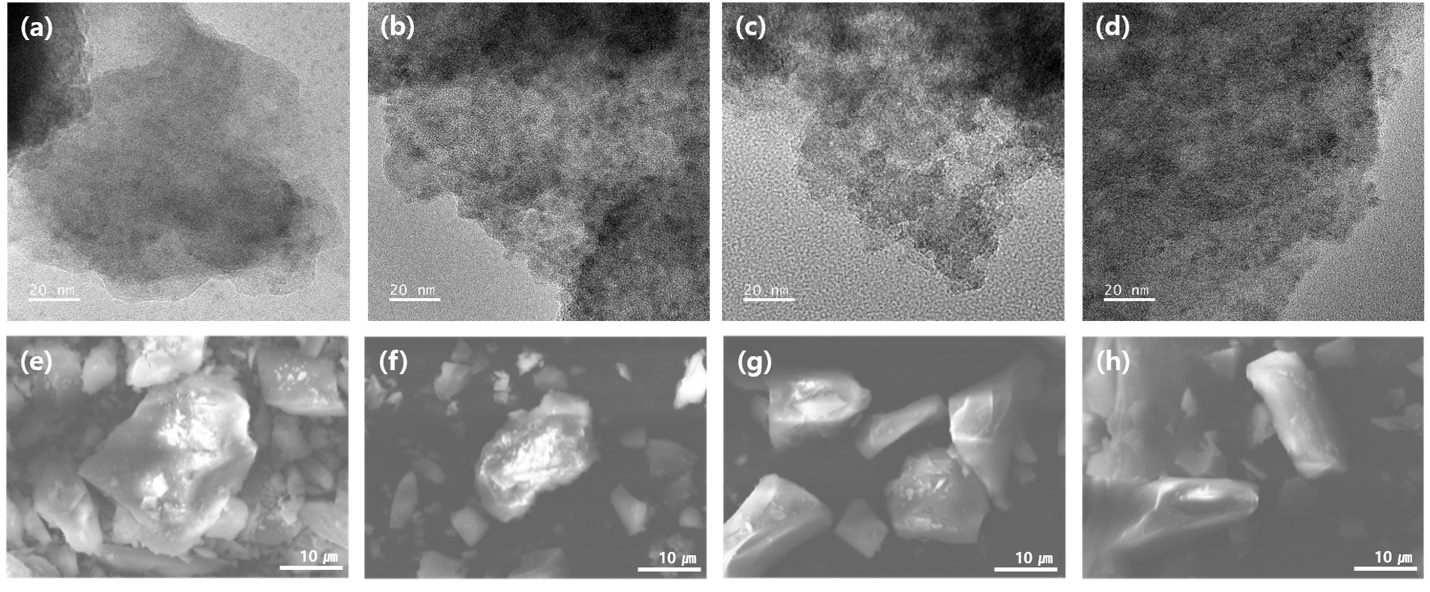


**Fig. S2.** TEM images of Al_2_O_3_ encapsulated powder QDs of (a) CGS/ZnS, (b) CGS/ZnS/Zr(i-PrO)_4_, (c) CIS/ZnS and (d) CIS/ZnS/Zr(i-PrO)_4_. SEM images of Al_2_O_3_ encapsulated QDs hybrid powders of (e) CGS/ZnS, (f) CGS/ZnS/Zr(i-PrO)_4_, (g) CIS/ZnS and (h) CIS/ZnS/Zr(i-PrO)_4_.


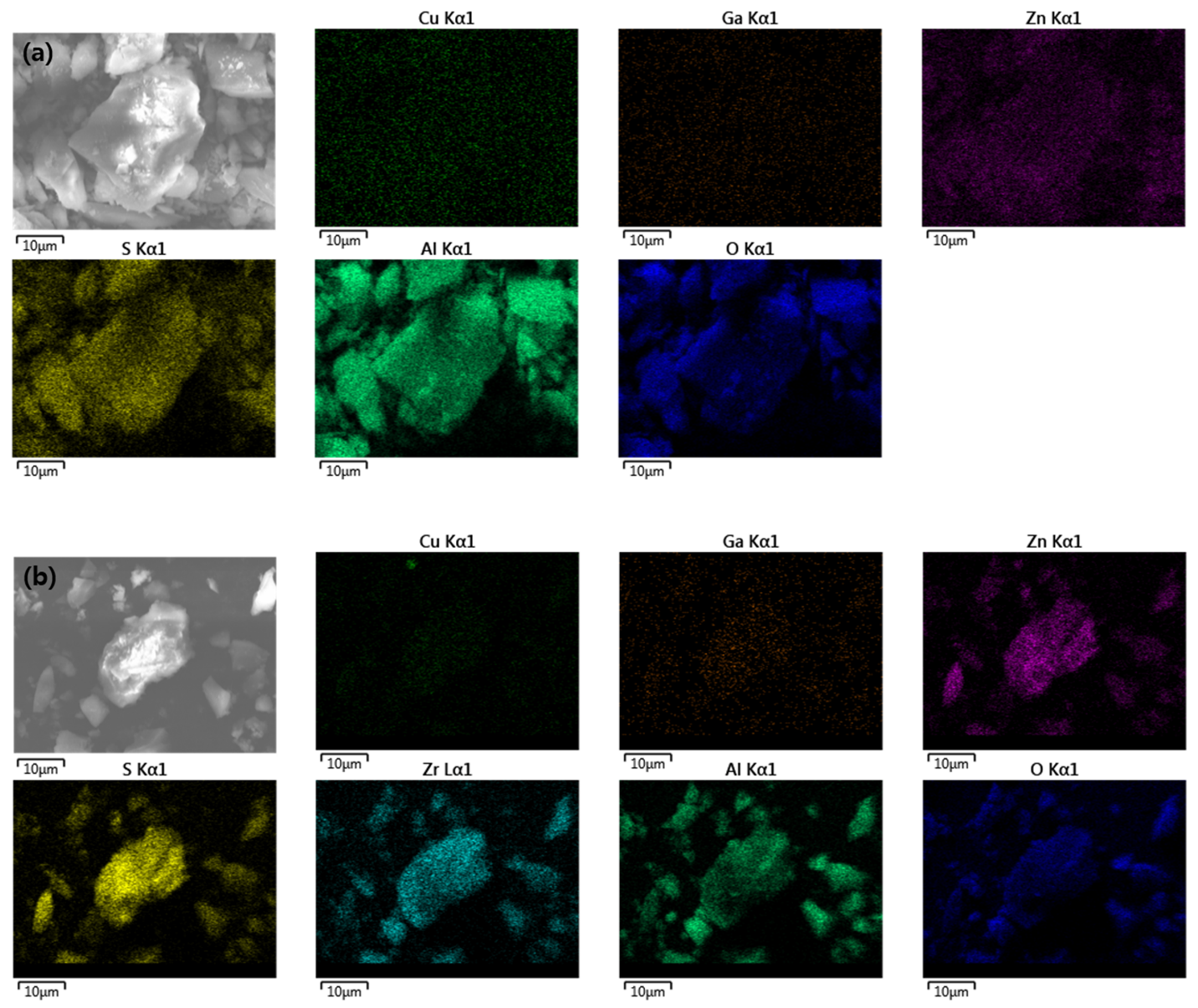


**Fig. S3.** SEM images and EDS data of (a) CGS/ZnS@Al_2_O_3_ and (b) CGS/ZnS/Zr(i-PrO)_4_@Al_2_O_3_ green QD hybrid powders.


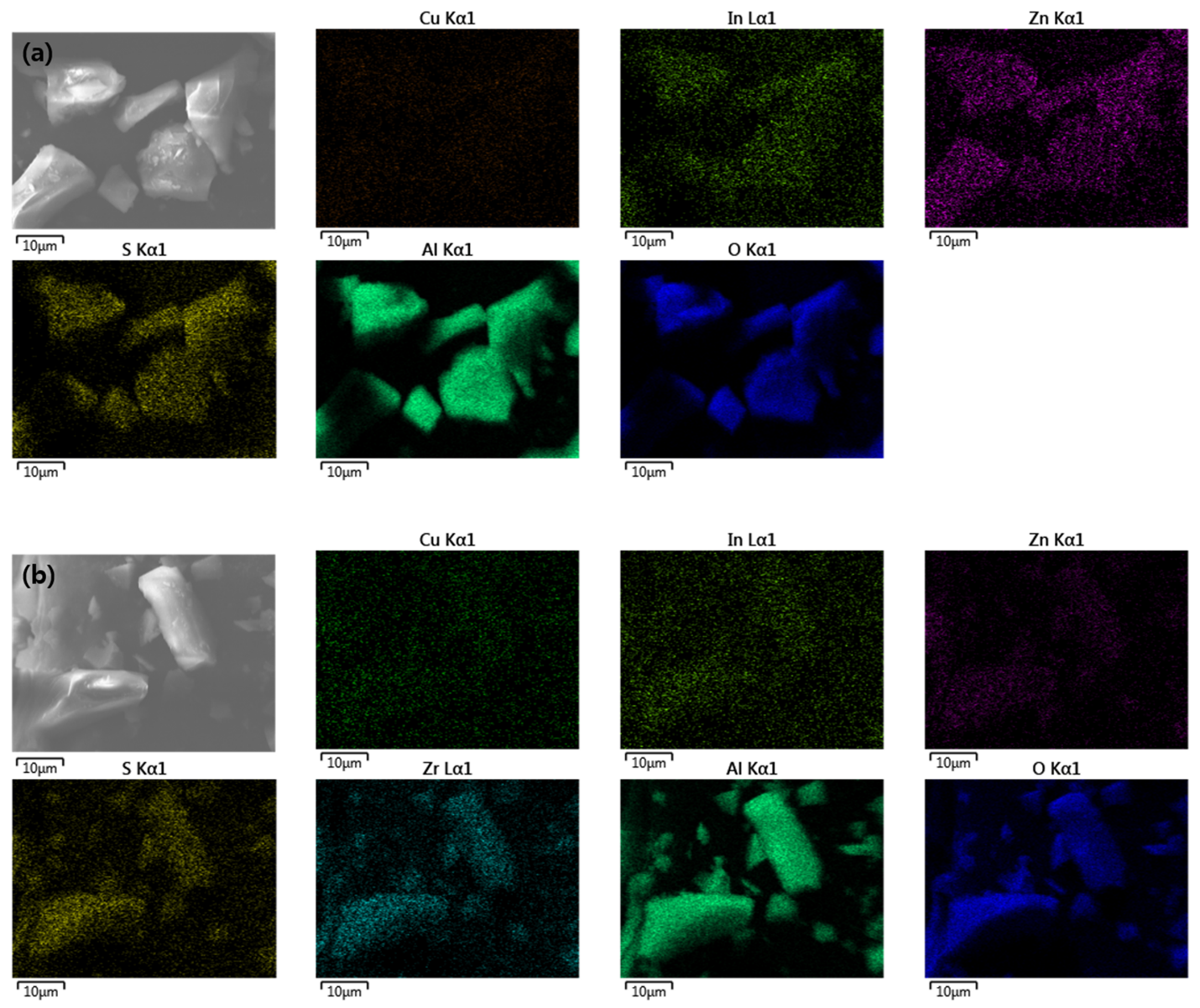


**Fig. S4.** SEM images and EDS mapping data of (a) CIS/ZnS@Al_2_O_3_ and (b) CIS/ZnS/Zr(i-PrO)_4_@Al_2_O_3_ red QD hybrid powders.


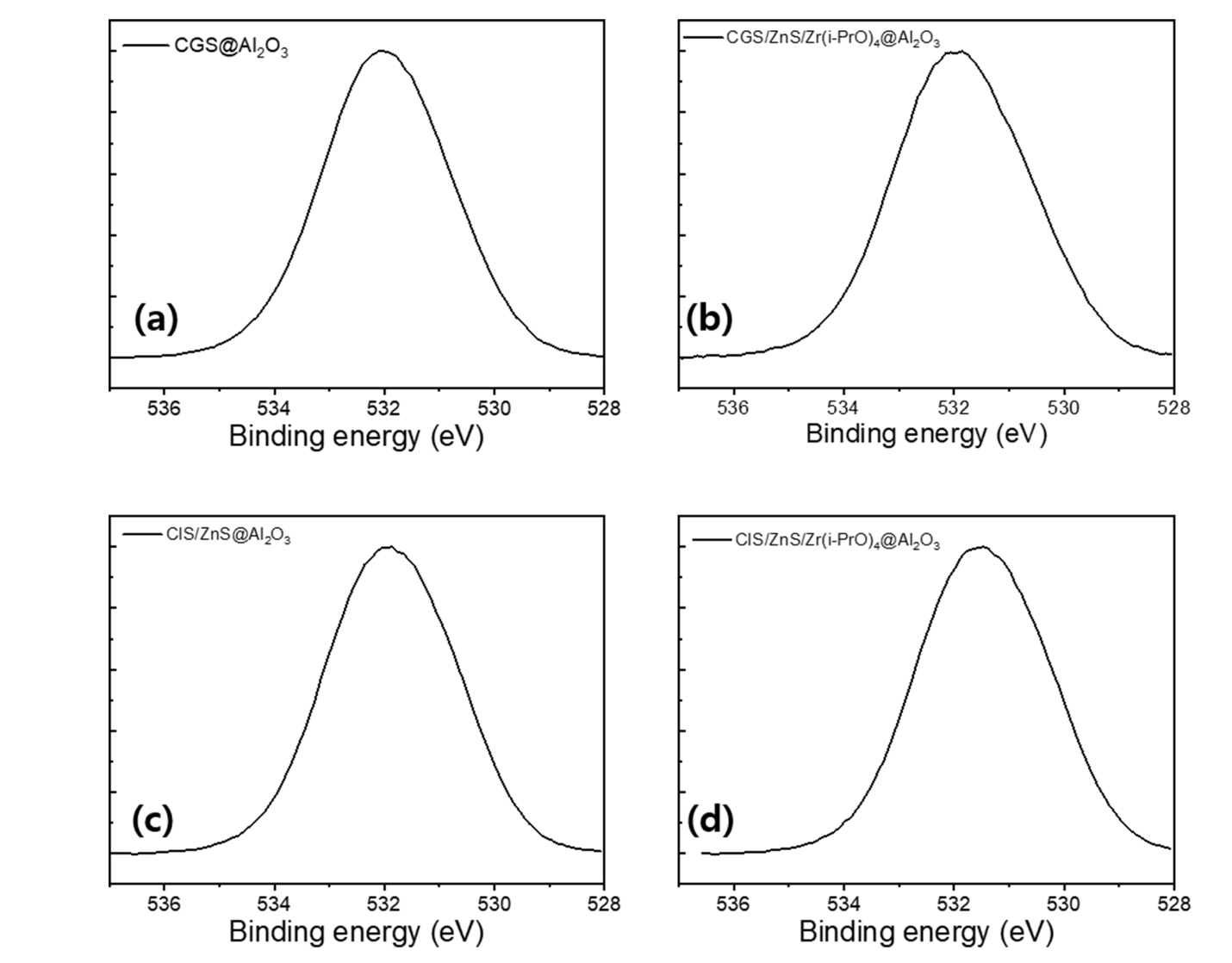


**Fig. S5.** Partial XPS survey results of O component peaks of Al_2_O_3_ encapsulated of green (a) CGS/ZnS, (b) CGS/ZnS/Zr(i-PrO)_4_ QDs, and red (c) CIS/ZnS, (d) CIS/ZnS/Zr(i-PrO)_4_, QDs.


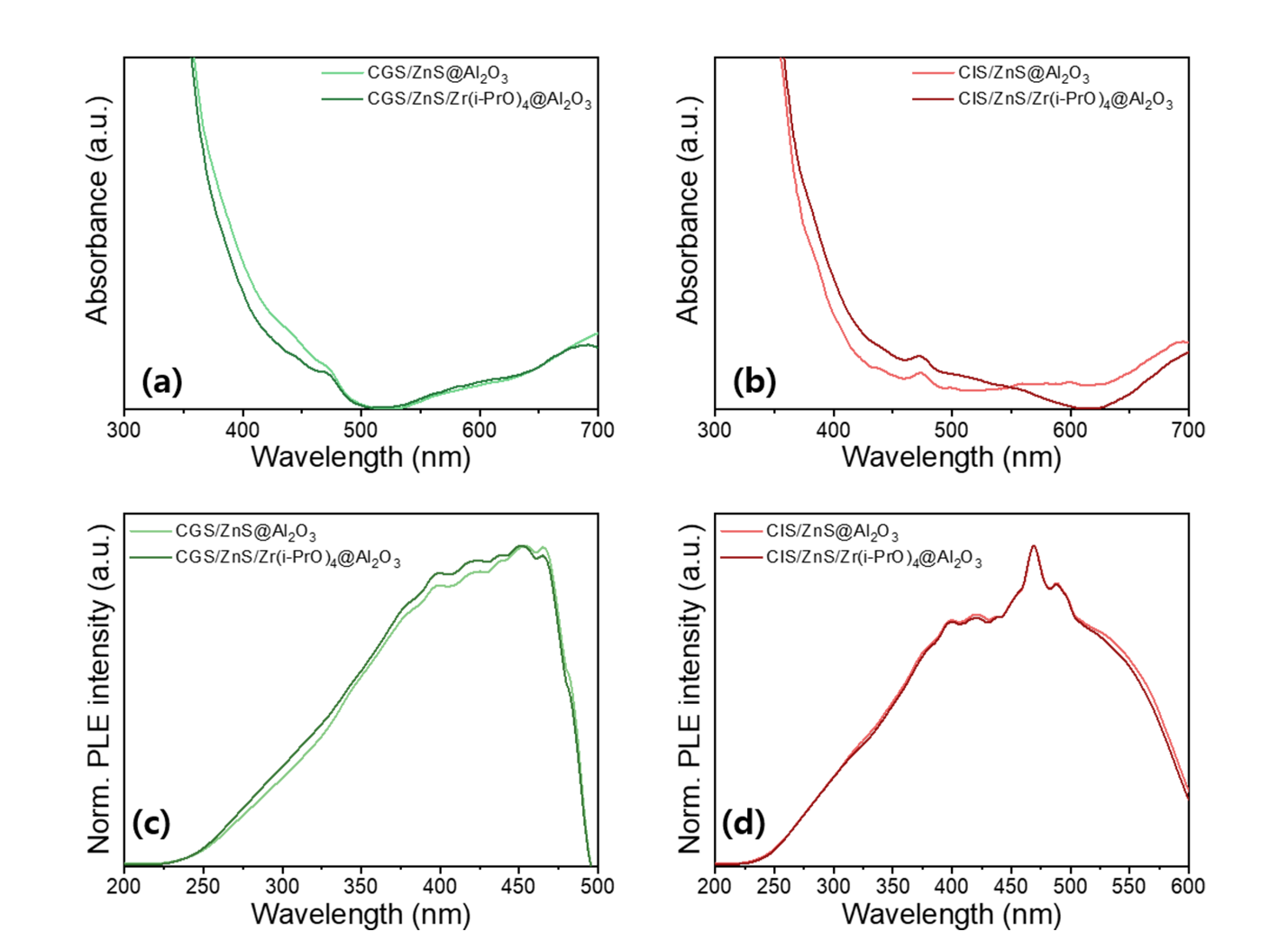


**Fig. S6.** Absorbance spectra of Al_2_O_3_ encapsulated of (a) green CGS/ZnS based powder QDs (b) red CIS/ZnS based powder QDs. And PLE spectra of Al_2_O_3_ encapsulated of (c) green CGS/ZnS based QD hybrid powders (d) red CIS/ZnS based QD hybrid powders.
